# Aberration methylation of miR-34b was involved in regulating vascular calcification by targeting Notch1

**DOI:** 10.18632/aging.101973

**Published:** 2019-05-25

**Authors:** Xiao Lin, Fuxingzi Li, Feng Xu, Rong-Rong Cui, Dan Xiong, Jia-Yu Zhong, Ting Zhu, Su-Kang Shan, Feng Wu, Xu-Biao Xie, Xiao-Bo Liao, Ling-Qing Yuan

**Affiliations:** 1 Department of Endocrinology and Metabolism, National Clinical Research Center for Metabolic Diseases, The Second Xiang-Ya Hospital, Central South University, Changsha, Hunan, People’s Republic of China; 2 Department of Geriatrics, Institute of Aging and Geriatrics, The Second Xiang-Ya Hospital, Central South University, Changsha, Hunan, People’s Republic of China; 3 Department of Endocrinology, Central Hospital of Yiyang, Yiyang, Hunan, People’s Republic of China; 4 Department of Pathology, The Second Xiang-Ya Hospital, Central South University, Changsha, Hunan, People’s Republic of China; 5 Center of Organ Transplantation, The Second Xiang-Ya Hospital, Central South University, Changsha, Hunan, People’s Republic of China; 6 Department of Cardiovascular Surgery, The Second Xiang-Ya Hospital, Central South University, Changsha, Hunan, People’s Republic of China; * Equal contribution

**Keywords:** miR-34b, vascular smooth muscle cells, vascular calcification, methylation, Notch1

## Abstract

Vascular calcification is one of the most important factors for cardiovascular and all-cause mortality in patients with end-stage renal diseases (ESRD). The current study was aimed to investigate the function and mechanisms of miR-34b on the calcification of vascular smooth muscle cells (VSMCs) both *in vitro* and *in vivo*. We found that the expression of miR-34b was significantly suppressed in VSMCs with high inorganic phosphate (Pi) treatment, as well as mouse arteries derived from 5/6 nephrectomy with a high-phosphate diet (0.9% Pi, 5/6 NTP) and human renal arteries from uraemia patients. Overexpression of miR-34b alleviated calcification of VSMCs, while VSMCs calcification was enhanced by inhibiting the expression of miR-34b. Bisulphite sequencing PCR (BSP) uncovered that CpG sites upstream of miR-34b DNA were hypermethylated in calcified VSMCs and calcified arteries due to 5/6 NTP, as well as calcified renal arterial tissues from uraemia patients. Meantime, increased DNA methyltransferase 3a (DNMT3a) resulted in the hypermethylation of miR-34b in VSMCs, while 5-aza-2′-deoxycytidine (5-aza) reduced the methylation rate of miR-34b and restored the expression of miR-34b in VSMCs. When DNMT3a was knocked down using DNMT3a siRNA, the effect of 3.5 mM of Pi on calcification of VSMCs was abrogated. In addition, Notch1 was validated as the functional target of miR-34b and involved in the process of calcification of VSMCs. Taken together, our data showed a specific role for miR-34b in regulating calcification of VSMCs both *in vitro* and *in vivo*, which was regulated by upstream DNA methylation of miR-34b and modulated by the downstream target gene expression, Notch1. These results suggested that modulation of miR-34b may offer new insight into a novel therapeutic approach for vascular calcification.

## INTRODUCTION

Vascular calcification is an independent predictor of cardiovascular events and associated with significant morbidity and mortality. Vascular calcification is common in diabetes mellitus [1], end stage renal disease (ESRD) [2] and the elderly [3], which is characterised by the maladaptive transdifferentiation of vascular smooth muscle cells (VSMCs) toward osteoblast-like cells and results in hydroxyapatite deposition and eventually mineralisation of the arterial wall [4, 5]. Therefore, the process of vascular calcification is similar to bone formation, which is an active and cell-regulated process. However, the mechanisms of vascular calcification are still poorly understood.

MicroRNAs (miRNAs), endogenous, single-stranded, small noncoding RNAs, negatively regulate gene expression post-transcriptionally via complementary base pairing to sequences within the 3′-untranslated regions (3′-UTR) of protein-coding mRNA transcripts, and they have potential as therapeutic targets [6]. Although miRNAs have been implicated in various cardiovascular biological processes including cell proliferation, differentiation, apoptosis and migration [7, 8], the roles of miRNAs in vascular calcification and the mechanisms involved are still largely unexplored. Notably, miR-34b played a critical role in the differentiation of osteoblasts [9, 10]. Moreover, Hao et al. reported that miR-34b was suppressed in aldosterone-induced VSMCs calcification [11]. Nevertheless, the mechanisms underlying miR-34b dysregulation during vascular calcification remains unknown.

DNA methylation, one of the most important epigenetic regulations, is mediated by DNA methyltransferases (DNMTs), including DNMT1, DNMT3a and DNMT3b [12]. A methyl group is added to the cytosine residue within cytosine phosphate-guanine (CpG) islands, often located on the first exon or promoter of genes [13]. The methylated CpGs then serve as a docking site for transcriptional repressors to down-regulate target gene transcriptions. Although several lines of evidence suggested that aberrant miRNAs were tightly linked to DNA methylation in cardiovascular diseases [14, 15], and Xie et al. reported that the expression of miR-34b was inversely correlated to CpG island methylation in hepatocellular carcinoma cancer [16]. However, whether DNA methylation could regulate the expression of miR-34b and influence vascular calcification has received little attention.

In the current study, we sought to explore the epigenetic mechanism of miR-34b suppression and its function in vascular calcification. We found that the notably reduced expression of miR-34b was due to hypermethylation of the promoter and then clarified that DNMT3a was responsible for hypermethylation of the promoter both *in vitro* and *in vivo*. While, 5-aza-2′-deoxycytidine (5-aza) reduced the level of DNMT3a, and thus, restored the expression of miR-34b in VSMCs, which resulted in an attenuation of calcification of VSMCs *in vitro*. Moreover, we also demonstrated that Notch1 was the target of miR-34b and involved in the process of VSMCs calcification. Thus, a novel epigenetic mechanism of miRNAs regulating VSMCs calcification was founded and provided a new insight into therapeutic potentials of vascular calcification.

## RESULTS

### miR-34b was down-regulated and involved in regulating osteoblastic differentiation of VSMCs

**Figure 1:**
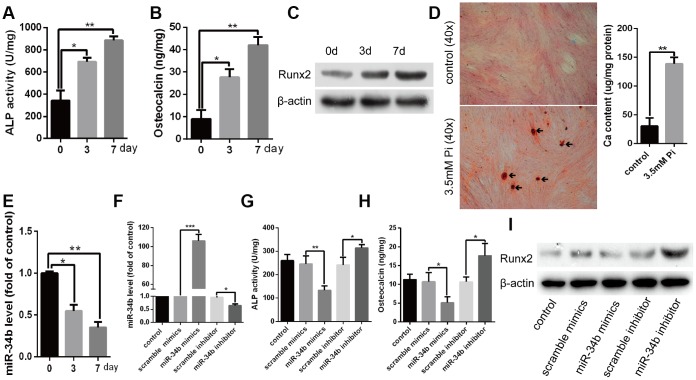
**MiR-34b was down-regulated and involved in the process of VSMCs calcification.** (**A**–**C**) VSMCs cultured in 3.5 mM of Pi were measured for ALP activity, OC secretion and Runx2 expression. (**D**) VSMCs were treated with 3.5 mM of Pi for 18 days and then subjected to Alizarin Red S staining. The calcium content was extracted with cetyl-pyridinium chloride and quantified by spectrophotometry. Representative images were shown. The arrows indicate the mineralised nodules. (**E**) Expression of miR-34b was detected by qRT–PCR in cultured VSMCs. (**F**) qRT–PCR showed the expression of miR-34b in VSMCs transfected with scramble miRNA mimics, inhibitor or miR-34b mimics, miR-34b inhibitor, respectively. (**G**–**I**) VSMCs were transfected to detect the change of ALP activity, OC secretion and Runx2 protein levels. n=3. The data were expressed as mean ± SD, *p < 0.05; **p < 0.005; ***p < 0.0005.

Firstly, we treated VSMCs with 3.5 mM of Pi to induce calcification of VSMCs and found that ALP activity, osteocalcin （OC） secretion and Runx2 were all significantly increased in a time-dependent manner when compared with the control (Figure 1A–1C). Moreover, Alizarin Red S staining verified that matrix mineral deposition was also significantly increased in VSMCs treated with 3.5 mM of Pi for 18 days (Figure 1D), while the expression of miR-34b was significantly down-regulated in VSMCs cultured with 3.5 mM of Pi (Figure 1E). These data suggested that miR-34b might be involved in regulating osteoblastic differentiation of VSMCs. To analyse whether miR-34b played a role in regulating osteoblastic differentiation of VSMCs, miR-34b mimics and an miR-34b inhibitor were transfected into VSMCs to overexpress miR-34b or inhibit the expression of miR-34b, respectively (Figure 1F). Interestingly, we found that overexpression of miR-34b significantly decreased ALP activity, OC secretion and Runx2 protein levels, whereas inhibiting the expression of miR-34b resulted in opposite results (Figure 1G–1I). These results indicated that miR-34b played a negative role in the calcification of VSMCs.

### The down-regulation of miR-34b was related with hypermethylation of DNA upstream of the CpG site during the process of VSMCs calcification

**Figure 2:**
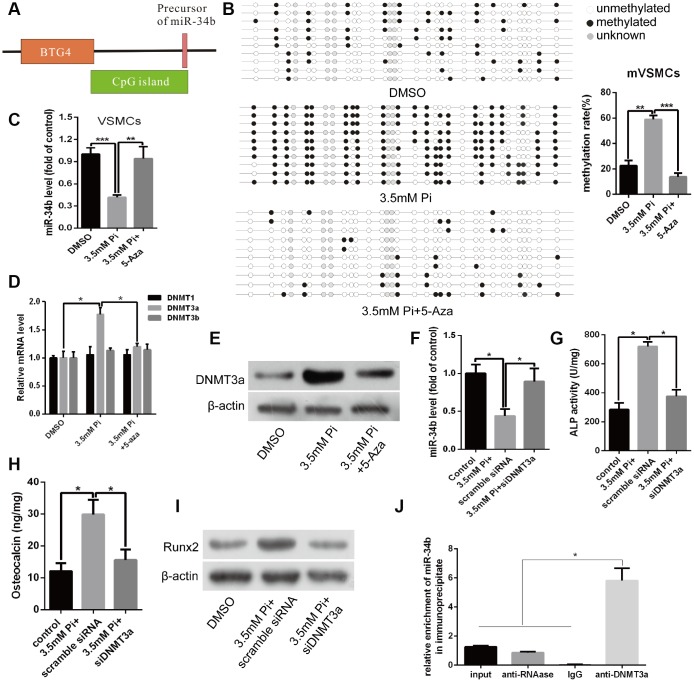
**Down-regulation of miR-34b was related with DNA upstream of CpG site methylation and mediated by DNMT3a that was involved in regulating VSMCs calcification.** (**A**) A schematic illustration of the location of the CpG islands upstream of miR-34b DNA. (**B**) BSP showed that the methylation rate of CpG sites of miR-34b DNA was significantly higher in VSMCs with 3.5 mM of Pi treatment than that of control. Meanwhile, 5-aza (10 μmol/L), a DNA methyltransferase inhibitor, decreased the methylation level of CpG sites of miR-34b in VSMCs. (**C**) The expression of miR-34b was detected by qRT-PCR in VSMCs treated with 3.5 mM of Pi or 3.5 mM of Pi + 10 μmol/L of 5-aza. (**D**) qRT-PCR showed the different expression levels of DNMT1, DNMT3a and DNMT3b in VSMCs cultured in 3.5 mM of Pi or 3.5 mM Pi + 10 μmol/L of 5-aza. (**E**) Western blot analysis showed that the different levels of DNMT3a protein in VSMCs treated with 3.5 mM of Pi or 3.5 mM Pi + 10 μmol/L of 5-aza. (**F**) qRT-PCR detected the expression of miR-34b after knocking down DNMT3a with DNMT3a siRNA in VSMCs. (**G**–**I**) ALP activity, OC secretion and Runx2 expression were determined in VSMCs induced by 3.5 mM of Pi after transfecting with scramble siRNA or DNMT3a siRNA. (**J**) Cultured VSMCs were subjected to ChIP using the anti-DNMT3a antibody, followed by qPCR analysis using primers annealing to the promoter sequence of miR-34b. n = 3. The data were expressed as mean ± SD, *p < 0.05; **p < 0.005; ***p < 0.0005.

Bioinformatics analysis revealed that the promoter of miR-34b is embedded within a typical CpG island (Figure 2A). Based on this, we hypothesised that miR-34b expression may be modulated by methylation statues. As expected, bisulphite sequencing PCR (BSP) analysis showed that high methylation of the promoter CpG sites of miR-34b, in VSMCs treated with 3.5 mM of Pi, reached as high as 58.22%, while the methylation rate of CpG sites of miR-34b in control VSMCs was about 21.38% (Figure 2B). Nevertheless, when VSMCs were treated with a DNA methyltransferase inhibitor, 5-aza, the methylation level of miR-34b greatly decreased (Figure 2B). Meanwhile, the down-regulated expression of miR-34b in calcified VSMCs was restored with 5-aza treatment (Figure 2C). Thus, we considered that the downregulation of miR-34b in calcified VSMCs was associated with DNA methylation.

The B-cell translocation gene 4 (BTG4), a member of the BTG family, is characterised by its antiproliferative properties. Previous studies reported that the CpG island of miR-34b was a bidirectional promoter, which drove the expression of both miR-34b and BTG4. Thus, methylation of CpG islands was also associated with transcriptional silencing of BTG4 [17]. In the present study, we also found that BTG4 mRNA, as well as proteins, were inhibited significantly in calcified VSMCs compared with the control, while 5-aza treatment rapidly restored the expression of BTG4 (Supplementary Figure 2A and 2B). These data suggested that down-regulation of miR-34b in calcified VSMCs was strongly associated with hypermethylation of CpG islands, which notably harboured bidirectional promoter activity, and regulated the expression of BTG4.

### DNMT3a mediated DNA methylation of miR-34b and involved in regulating osteoblastic differentiation of VSMCs

Next, we investigated whether the silencing of miR-34b, caused by DNA methylation, was involved in regulating osteoblastic differentiation of VSMCs. Previous studies demonstrated that DNA methylation was regulated by DNMTs, including DNMT1, 3a and 3b [18]. Accordingly, an obvious increase in the expression of DNMT3a mRNA was observed by qRT- PCR in calcified VSMCs. However, neither DNMT1 nor DNMT3b were significantly changed between calcified and control VSMCs (Figure 2D). Additionally, the level of DNMT3a protein agreed with the expression of mRNA (Figure 2E). However, treating VSMCs with 5-aza remarkably decreased the expression of DNMT3a at both the mRNA and protein levels (Figure 2D and 2E). Furthermore, by knocking down the expression of DNMT3a via transfecting DNMT3a siRNA into VSMCs, the effect of 3.5 mM of Pi on reducing the expression of miR-34b was abolished (Figure 2F). Three independent sequences of siDNMT3a were verified in our current experiment and the second siDNMT3a was the most effective (Supplementary Figure 2C). In addition, the effects of a high Pi concentration on the expression of osteoblastic markers including ALP activity and OC secretion, as well as Runx2, were also diminished when DNMT3a was knocked-down (Figure 2G–2I). Moreover, the methylation rate of CpG sites of miR-34b DNA was significantly lower in VSMCs when DNMT3a was knocked-down (Supplementary Figure 2D). Additionally, the chromatin immunoprecipitation (ChIP) analysis verified the presence of the miR-34b sequence in chromatin samples, pulled down by anti-DNMT3a, and confirmed the binding of DNMT3a to the promoter of miR-34b (Figure 2J). Taken together, knocking-down DNMT3a completely blocked the effects of a high Pi concentration on the calcification of VSMCs and restored the expression of miR-34b, which suggested that the miR-34b-regulated VSMCs calcification was dependent on the expression of DNMT3a.

### Notch1 was the direct target of miR-34b and involved in regulating VSMCs calcification

**Figure 3:**
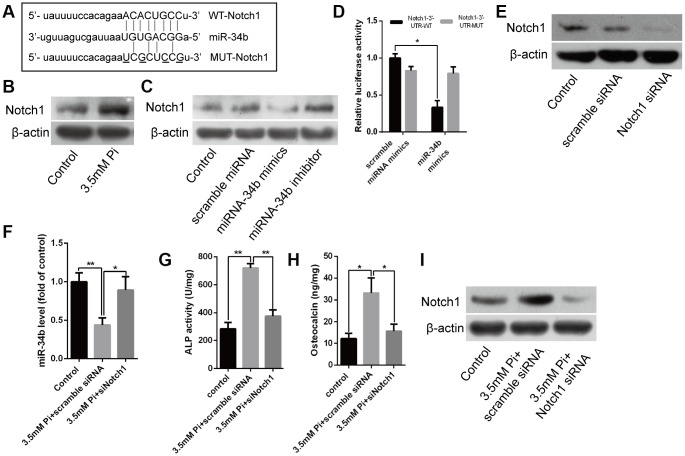
**Notch1 was the direct target of miR-34b and involved in regulating VSMCs calcification.** (**A**) Schematic of the miR-34b putative target sites in mouse Notch1 3′-UTR and alignment of miR-34b with WT and MUT Notch1 3′-UTR, showing pairing. The mutated nucleotides were underlined. (**B**) Western blot analysis showed the level of Notch1 protein in VSMCs treated with 3.5 mM of Pi. (**C**) VSMCs were transfected with scramble miRNA mimics, inhibitor or miR-34b mimics, miR-34b inhibitor, respectively, and harvested for the examination of Notch1 protein by western blot analysis. (**D**) The WT-Notch1 3′-UTR and the MUT- Notch1 3′-UTR reporters were co-transfected with miR-34b mimics or control oligos into VSMCs. Forty-eight hours after transfection, the luciferase activities were measured. (**E**) Notch1 was knocked down successfully by Notch1 siRNA. (**F**) The expression of miR-34b was assessed by qRT-PCR in VSMCs induced by 3.5 mM of Pi after transfecting with scramble siRNA or Notch1 siRNA. (**G**–**I**) ALP activity, OC secretion and Runx2 expression were determined in VSMCs induced by 3.5 mM of Pi after transfecting with scramble siRNA or Notch1 siRNA. n = 3. The data were expressed as mean ± SD, *p < 0.05; **p < 0.005.

Bioinformatics analysis showed that Notch1 was one of the putative targets of miR-34b (Figure 3A). Interestingly, western blot analysis verified that the levels of Notch1 protein were significantly higher in VSMCs treated with 3.5 mM of Pi (Figure 3B), which indicated a negative correlation between the expression of miR-34b and Notch1. Further, overexpression of miR-34b, by transfecting miR-34b mimics into VSMCs, significantly reduced the expression of Notch1, while the miR-34b inhibitor moderately increased the expression of Notch1 (Figure 3C). Moreover, a luciferase reporter construct containing the wild-type (WT) or mutant 3′-UTR coding sequences for Notch1 was introduced with miR-34b mimics into VSMCs, and the overexpression of miR-34b significantly decreased the relative luciferase activity of the WT-3′-UTR of Notch1 reporter plasmids, but the inhibitory effect of miR-34b on the relative luciferase activity was abrogated after Notch1 mRNA 3′-UTR was mutated. Scramble miRNAs mimics, however, did not affect the WT or mutant constructs (Figure 3D). Lastly, Notch1 was knocked down successfully in VSMCs by siRNA technology (Figure 3E) and the effect of 3.5 mM of Pi on the expression of miR-34b was abrogated (Figure 3F). Intriguingly, the effect of 3.5 mM of Pi to on inducing calcification of VSMCs was also diminished when Notch1 was knocked down, which was confirmed by the ALP activity level, OC secretion and Runx2 were similar to the control (Figure 3G–3I). Collectively, these results suggested that miR-34b modulated VSMCs calcification by directly targeting Notch1.

### miR-34b inhibited vascular calcification in mice

**Figure 4:**
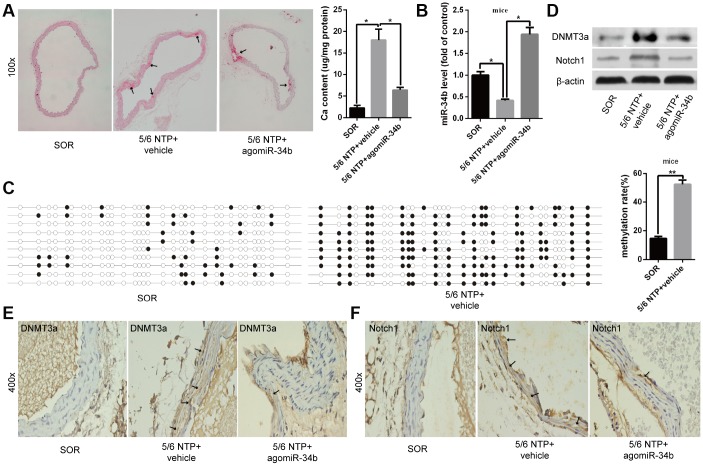
**MiR-34b inhibited vascular calcification in mice.** (**A**) Alizarin Red S staining showed calcified arteries in 5/6 NTP+vehicle mice but not SOR mice, and calcification was attenuated in 5/6 NTP+agomiR-34b mice. n = 5. Representative images were shown. The arrows indicate calcification sites in the mouse artery. (**B**) qRT–PCR showed the expression of miR-34b in SOR group mice, 5/6 NTP+vehicle mice and 5/6 NTP+agomiR-34b mice, respectively. (**C**) BSP showed the methylation rate of CpG sites of miR-34b DNA in arteries from 5/6 NTP+vehicle mice, was significantly higher than that from SOR arteries. n = 3. Representative images were shown. (**D**–**F**) The levels of DNMT3a and Notch1 were determined by western blot and immunohistochemistry staining analysis among arteries from the SOR group, 5/6 NTP+vehicle mice and 5/6 NTP+agomiR-34b mice, respectively. The data were expressed as mean ± SD, *p < 0.05. SOR: sham operation; 5/6 NTP: 5/6 nephrectomy with a high-phosphate diet.

To illuminate the role of miR-34b regulating vascular calcification *in vivo*, 5/6 nephrectomy with a high-phosphate diet (5/6 NTP) mice was used as a vascular calcification model. Alizarin Red S staining showed that aortas derived from 5/6 NTP+vehicle mice displayed extensive calcification compared with those from the sham operation (SOR) group mice (Figure 4A). At the same time, the osteoblastic differentiation markers, including ALP, OC and Runx2, were significantly increased in calcified aortas (Supplementary Figure 3). However, vascular calcification was significantly alleviated in the aortas from 5/6 NTP+agomiR-34b-treated mice, which was verified by decreased mineral deposition, as well as ALP, OC and Runx2 expression (Figure 4A and Supplementary Figure 3). Meanwhile, qRT–PCR analysis revealed that the expression of miR-34b was decreased in calcified mice aortas from 5/6 NTP+vehicle mice but markedly elevated in those from 5/6 NTP+agomiR-34b-treated mice (Figure 4B). In accordance with these results, the BSP assay demonstrated that the methylation rate of miR-34b in calcified mouse arterial tissues, isolated from 5/6 NTP+vehicle mice, reached 56.89% (Figure 4C). Moreover, both western blotting and immunohistochemistry staining analysis showed an obvious increase of DNMT3a in calcified aortic tissues in 5/6 NTP+vehicle mice when compared with SOR mice (Figure 4D and 4E). Last but not least, the expression of the miR-34b target gene, Notch1, also increased significantly in calcified aortas from 5/6 NTP+vehicle mice but decreased greatly in that from 5/6 NTP+agomiR-34b-treated mice (Figure 4D and 4F). Taken together, these data suggested that miR-34b inhibited vascular calcification through targeting Notch1 *in vivo* and the down-regulation of miR-34b in calcified aortas was regulated by hypermethylation.

### Testing the function of miR-34b in arteries from patients with uraemia

**Figure 5:**
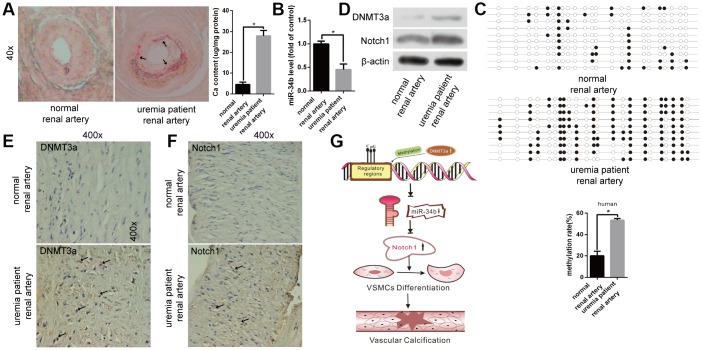
**The function of miR-34b in patients with uraemia.** (**A**) Alizarin Red S staining showed normal renal arteries from healthy donors and calcified renal arteries from uraemia patients. n = 5. Representative images were shown. The arrows indicate the calcification sites of the human renal artery. (**B**) qRT-PCR detected the expression of miR-34b in arteries from healthy donors and uraemia patients, respectively. (**C**) The methylation rate of CpG sites of miR-34 DNA in arteries from uraemia patients was significantly higher than that from normal donors. n = 3. Representative images were shown. (**D**–**F**) The levels of DNMT3a and Notch1 were determined by western blot and immunohistochemistry staining analysis in arteries from healthy donors and uraemia patients. (**G**) The proposed model of miR-34b regulating the process of vascular calcification. The data were expressed as mean ± SD, *p < 0.05.

Lastly, we examined the difference in the miR-34b levels between human renal arteries from uraemia patients and normal renal arteries from healthy donors. Arterial calcification was confirmed by Alizarin Red S staining in renal arterial tissues from uraemia patients (Figure 5A), while the expression of miR-34b was found to be significantly lower in calcified renal arteries than that in normal arteries (Figure 5B). Meanwhile, we found that the methylation rate of miR-34b in the calcified renal arteries from uraemia patients was much higher than that from normal subjects according to BSP analysis (Figure 5C). Moreover, the levels of DNMT3a protein, as well as Notch1 protein, were also increased in renal arteries from uraemia patients, which was proved by both western blotting analysis and immunohistochemistry staining (Figure 5D–5F). Thus, these data suggested that miR-34b regulated vascular calcification in uraemia patients.

## DISCUSSION

In the present study, we found that miR-34b was significantly down-regulated in VSMCs with high Pi treatment and acted as a negative regulator during osteoblastic differentiation of VSMCs both *in vitro* and *in vivo*. Moreover, we first clarified that the down regulation of miR-34b was associated with its upstream DNA methylation and regulated by DNMT3a. Furthermore, Notch1 was verified as the direct target of miR-34b and involved in modulating vascular calcification. The proposed mechanism by which miR-34b regulates the process of vascular calcification is shown in Figure 5G.

Vascular calcification, a well-regulated process that involves the transdifferentiation of VSMCs into osteoblast-like cells, is a pathology that often occurs in the elderly and patients with diabetes mellitus and ESRD [1, 2, 19]. In accordance with these studies, the Alizarin Red S staining results in our study showed that the renal arterial tissues collected from uraemia patients were severely calcified. Though numerous studies had demonstrated that miRNAs played an important role in regulating vascular calcification [20, 21], the specific mechanisms involved still needed to be elucidated. Another important miRNA, miR-34b, also played important roles in skeletal and muscle development, physiology, and disease pathogenesis [22, 23]. For example, Wei et al. found that miR-34b inhibited osteoblast proliferation and differentiation in mice by targeting Satb2 [9]. Data from Bae showed that miR-34b/c played a critical role in bone homeostasis, in part, through modulating Notch signalling both *in vitro* and *in vivo* [23]. Recently, Hao et al. reported that miR-34b was suppressed in aldosterone-induced VSMC calcification [11]. However, the mechanisms underlying miR-34b dysregulation during vascular calcification remains unknown. In our present study, we also found that miR-34b was down-regulated both in VSMCs induced by a high Pi concentration and arterial tissues from 5/6 NTP, as well as uraemia patients. Moreover, overexpression of miR-34b both in VSMCs and in mice could attenuate vascular calcification.

Recently, epigenetic modifications—including DNA methylation, histone modifications, acetylation and miRNAs—have become an important and crucial method for regulating gene expression [24]. Hu et al. showed that miR-1298 was regulated by DNA methylation and affected the proliferation and migration of VSMCs by targeting connexin 43 [14]. Another study showed that miR-143 was hypermethylated, which was regulated by DNMT3a in Hcy-induced proliferation of VSMCs [22, 25]. Our previous study also demonstrated that the methylation level of miR-204 was regulated by DNMT3a in the process of arterial calcification [26]. Interestingly, previous studies had shown that the upstream DNA features of miR-34b were embedded within a typical CpG island and shared the same promoter with the BTG4 gene [17, 27]. Therefore, we assumed that miR-34b might be regulated by methylation. Firstly, we found a potentially functional site in the promoter region of miR-34b (423-bp upstream from the transcription start site) via silico searching and database mining, which located in a typical CpG island. Accordingly, the results showed that upstream CpG sites of miR-34b DNA were hypermethylated both in calcified VSMCs and arterial tissues. Meanwhile, both BTG4 mRNA and protein levels decreased significantly in calcified VSMCs compared with the control because the CpG site of miR-34b DNA was a bidirectional promoter, which drives the expression of both miR-34b and BTG4 [17]. Interestingly, treating VSMCs with 5-aza, a DNA methyltransferase inhibitor, decreased the methylation level of miR-34b markedly and rapidly restored expression of miR-34b and BTG4. These data suggested that the down-regulation of miR-34b in the process of vascular calcification was strongly associated with hypermethylation of its neighbouring CpG island. However, whether the expression of miR-34b was mediated by methylation in 5/6 NTP mice and whether 5-aza has a similar role in mice as it does in VSMCs requires further investigation.

It is well known that DNA methylation is usually mediated by a series of DNMTs, such as DNMT1, 3a and 3b [18, 28]. Azechi et al. reported that upregulation of ALP expression along, with a reduction in the DNA methylation level of the ALP promoter region, facilitated the Pi-induced vascular calcification, which was achieved by the downregulation of DNMT1 expression [29]. Recently, Xie et al. demonstrated that DNMT1 negatively regulated arterial stiffening via maintaining the contractile phenotype of VSMCs [30]. In addition, our previous study also clarified that DNMT3a was involved in regulating the differentiation of VSMCs [26]. Based on these data, our results showed that both the mRNA and protein level of DNMT3a, but not DNMT1 or DNMT3b, were significantly increased in VSMCs treated with a high Pi concentration. Moreover, an obvious increase in the expression of DNMT3a was observed in arterial tissues derived from 5/6 NTP mice and uraemia patients. DNMT1 contributed to the maintenance of DNA methylation patterns, whereas DNMT3a and DNMT3b promoted methylation at previously unmethylated CpG sites [18]. This might explain why only DNMT3a was changed during the process of vascular calcification. However, VSMCs treated with 5-aza had a remarkable decrease in the expression of DNMT3a protein levels and BSP, showing that the methylation level of miR-34b in VSMCs treated with 5-aza was much lower. Thus, the expression of miR-34b was restored rapidly. Furthermore, knocking down DNMT3a blocked the effect of 3.5 mM of Pi on the differentiation of VSMCs, which suggested that the increased expression of DNMT3a resulting in the hypermethylation of miR-34b and DNMT3a played a crucial role in regulating VSMCs calcification. However, whether DNMT3a also has a key role *in vivo* needs further study.

Numerous studies have demonstrated that miRNAs usually regulate genes expression through binding to the 3′-UTR sites of their target genes [20, 26]. Notch1 is a transmembrane protein that plays a critical role in the determination of cellular proliferation, apoptosis and differentiation, especially in osteoblasts [23, 31–33]. For example, Yangjin Bae et al. demonstrated that miRNA-34b/c are up-regulated and directly target Notch1 during BMP2-induced C2C12 osteoblast differentiation [23]. Another study reported that Notch positively regulated osteoblastic cell differentiation [34]. Furthermore, Notch signalling could induce osteogenic differentiation and mineralisation of VSMCs [35]. Accordingly, in the present study, we also showed that Notch1 was expressed in VSMCs and upregulated in both calcified VSMCs and arteries. Moreover, we demonstrated that Notch1 was a novel target of miR-34b by showing that: (i) the Notch1 3′-UTR sequence contained a sequence that was complementary to the seed sequence of miR-34b; (ii) the overexpression of miR-34b significantly down regulated the expression of Notch1, whereas the functional inhibition of miR-34b resulted in the upregulation of Notch1 expression in VSMC; (iii) luciferase reporter assays confirmed that the overexpression of miR-34b mediated by the inhibition of Notch1 3′-UTR luciferase reporter activity was abolished by the insertion of mutations into the miR-34b seed binding site. In addition, the effects of a high Pi concentration on osteoblastic differentiation of VSMCs were completely blocked with the knock down of Notch1. Collectively, these results confirmed the view that miR-34b inhibited VSMC calcification by down-regulating Notch1, and that Notch1 was involved in regulating VSMCs calcification. These results are consistent with a recent study that also found that Notch1 was one of the target genes of miR-34b-5p in thyroid carcinoma [36]. However, though the expression of Notch1 was upregulated in both arteries from 5/6NTP mice and uraemia patients, the *in vivo* role of Notch1 in vascular calcification still needs to be studied further.

In summary, our results showed for the first time, to the best of our knowledge, that down-regulation of miR-34b was related to its DNA upstream hypermethylation of CpG sites in both calcified VSMCs and arteries. The down-regulated miR-34b was a novel regulator for differentiation of VSMCs and arteries by directly targeting Notch1. Therefore, these findings provided new insights into therapeutic strategies for vascular calcification and related cardiovascular diseases.

## MATERIALS AND METHODS

### Cell culture and transfection

VSMCs were isolated from 6–8 week-old male C57/BL mice as described before [21]. The VSMCs phenotype was verified by positive immunostaining of α-smooth muscle actin (α-SMA) (Supplementary Figure 1). VSMCs were seeded in DMEM supplemented with 10% FBS (Gibco BRL Co. New York, USA), penicillin (100 U/mL), and streptomycin (100 μg/mL) at 37 °C in a humidified atmosphere of 5% CO_2_. To induce calcification, 70% confluent VSMCs at passage 3–8 were cultured in DMEM supplemented with 3.5 mM of inorganic phosphate (3.5 mM Pi, NaH_2_PO_4_:Na_2_HPO_4_ = 1:2, pH = 7.0). For transient transfection of miR-34b mimics, inhibitors or Notch1 and DNMT3a siRNA oligos, a combination of oligos (50 nM) and Lipofectamine 2000 (Invitrogen Co.) were mixed following the manufacturer’s instructions and added to cells in 6-well plates at a density of 2 × 10^5^ cells per well. MiR-34b mimics, inhibitors and their control oligos, as well as miR-34b agomiR were purchased from Ribobio (Guangzhou, China). DNMT3a and Notch1 siRNA and control siRNA oligos were also purchased from Ribobio (Guangzhou, China).

### Alizarin Red S staining

Alizarin Red S staining was performed as previously described [37]. Briefly, for VSMCs, cells cultured with 3.5 mM of Pi for 18 days were fixed in 4% paraformaldehyde and then stained with 2% (pH 8.3) Alizarin Red S for 30 minutes at 37 °C. For artery samples, arteries were processed using the paraffin-embedded method. and subsequently, deparaffinised and rehydrated. Next, the artery sections were stained with 2% (pH 8.3) Alizarin Red S for 5 minutes at 37°C. The stained matrixes were assessed and photographed using a digital microscope. For the quantification of calcium levels, cells and arteries were washed with PBS and decalcified with 0.6 N HCl for 24 hours, calcium content was determined by measuring the concentrations of calcium in the HCl supernatant by atomic absorption spectroscopy. After decalcification, the cells were washed three times with PBS and the cells were solubilised with 0.1 N NaOH/0.1% SDS. The protein content was measured with a BCA protein assay. The calcium content of the cell layer was normalised to the protein content.

### Western blot analysis

Western blot analysis was carried out as previously described [38]. Briefly, proteins were extracted from VSMCs, mice or human arterial tissues. The protein was quantified using the BCA rotein kit (Beyotime Biotechnology, Shanghai, China). 30 μg of protein was loaded onto sodium dodecyl sulphate-polyacrylamide gel electrophoresis (SDS-PAGE) and transferred to polyvinylidene fluoride (PVDF) a membrane (Millipore, Billerica, USA). After blocking with 5% non-fat milk, the membrane was incubated with primary antibodies overnight at 4 °C. Antibodies including anti-ALP (ab83259, 1:1000), anti-OC (ab133612, 1:1000), anti-Runx2 (ab23981, 1:1000), anti-DNMT3a (3598, 1:500), anti-BTG4 (sc-323518, 1:1000), anti-Notch1 (ab52627, 1:1000) and anti-β-actin (AP53385, 1:3000). The next day, the membranes were washed with PBS-T and then incubated with horseradish peroxidase-conjugated goat-anti-rabbit (sc-2004, 1:5000) or horseradish peroxidase-conjugated goat-anti-mouse (sc-2005, 1:5000) secondary antibodies. The immunoreactive bands were detected using an ECL kit (Amersham Biosciences U.K. Ltd) and then analysed by Image-Pro Plus 6.0 software. The relative expression level of the target protein was normalised to the intensity of the β-actin band.

### Quantitative reverse transcription-polymerase chain reaction (qRT-PCR)

Total RNA was extracted from cultured VSMCs, mouse or human arterial tissues using Trizol Reagent (Invitrogen) [26]. All-in-One™ first-strand cDNA synthesis kit (AORT-0060, Genecopoiea) was used and the reverse-transcription reaction was carried out for 10 minutes at 65 °C, followed by a second step of 60 minutes at 37 °C, 5 minutes at 85 °C and a final hold at 4 °C. To detect the expression of genes mRNAs, All-in-One™ qPCR Mix (QP003, Genecopoiea) was carried out in a LightCycler® 96 System (Roche, Indianapolis, USA). The reactions were performed at 95 °C for 10 minutes, followed by 40 cycles of 95 °C for 10 seconds, 62 °C for 20 seconds and 72 °C for 15 seconds. Data was normalised to GAPDH values. The PCR primers purchased from Genecopoiea were as follows: DNMT1 (MQP029034), DNMT3a (MQP026954), DNMT3b (MQP023625), BTG4 (MQP031882) and GAPDH (MQP027158). For miR-34b analysis, the All-in-One™-miRNA-qRT-PCR detection system was used (AOMD-Q060, Genecopoiea) as described by the manufacturer’s protocol and using U6 snRNA as the reference. Briefly, a 25 μl reverse-transcription reaction was carried out for 60 minutes at 37 °C, 5 minutes at 85°C and a hold at 4 °C. qPCR was performed for 10 minutes at 95°C, followed by 40 cycles of 10 seconds at 95 °C, 20 seconds at 65 °C and 10 seconds at 72 °C. The primers for human miR-34b (HmiRQR0042), mice miR-34b (MmiRQP0980) and U6 snRNA (HmiRQP9001 and MmiRQP9002) were used. The relative standard curve method (2^-△△CT^) was used to determine the relative mRNA and miRNA expression. The results were expressed as fold change relative to the relevant control. The qPCR were run in three independent experiments and the results were presented as the mean ± standard error of samples.

### Methylation analysis using BSP

BSP was conducted as described previously [39]. The bisulphite-treated human miR-34b containing 23 CpG sites and mice miR-34b containing 32 CpG sites was amplified with the primers listed in Supplementary Table 1. The amplified PCR products were purified and subcloned into the pGM T-Easy vector (Promega, Madison, WI, USA) and a total of 10 clones of each sample were sequenced. The percentage of methylated CpG dinucleotides was calculated to evaluate the methylation level of miR-34b.

### ChIP and qRT-PCR

VSMCs were crosslinked, by incubation in formaldehyde, and then incubated with glycine to quench formaldehyde. ChIP assays were undertaken using the EZ-ChIP^TM^ Chromatin Immunoprecipitation Kit (#17-371, Millipore, USA) and an anti-DNMT3a antibody (ab2850, Abcam) following the manufacturer’s protocol. DNA in the chromatin was immunoprecipitated by the anti-DNMT3a antibody, anti-RNA Polymerase II or an IgG were subjected to qRT-PCR analysis of the mouse miR-34b promoter sequence. The primer sequences were as follows: forward 5′- GCTCTAGACTTGGGTCTGGAAGC-3′ and reverse 5′- CAGTGGAGTTAGTGATTGTCAGCAC-3′. To analyse the results, the percentage input method was used.

### Luciferase reporter assay

A segment of the 3'-UTR of mouse Notch1 with the predicted binding sites of miR-34b was cloned into XbaI-FseI restriction sites of the pGL3 luciferase reporter vector (Promega). The QuikChange site-directed mutagenesis kit (Stratagene) was employed to construct a mutant 3'-UTR of Notch1. Then, VSMCs were co-transfected with a luciferase reporter carrying wild type Notch1 3'-UTR (WT-pGL3-Notch1) and mutant Notch1 3'-UTR (MUT-pGL3-Notch1) and miR-34b mimics or scramble oligos, respectively. Forty-eight hours after transfection, luciferase activities were quantified with the luciferase assay system (Promega). The nucleotide sequences of primers for the construct and mutation of 3'-UTR Notch1 mRNA were purchased from Ribobio. (Guangzhou, China).

### Animals

Twenty-four 6-week-old male C57/BL/6 mice were randomly divided into three groups: SOR, 5/6 NTP+vehicle and 5/6 NTP+agomir-34b. The 5/6 NTP+agomir-34b group received 20 mg/kg body weight of agomir-34b and the 5/6 NTP+vehicle received the same volume vehicle through tail vein injection. The 5/6 NTP mouse model was set up as described in our previous study [26]. Briefly, the mice were anesthetised with an intraperitoneal injection of pentobarbital sodium (50 mg/kg). Then, the upper and lower poles of the left kidney were resected. Two weeks later, the right kidney was then removed completely. SOR mice underwent the same surgery over the same period as the 5/6 NTP mice, but only the renal capsule was stripped after exposure of the kidney and then the abdomen was closed. To accelerate the process of aortic calcification, animals were fed with a high-phosphate diet (0.9% Pi) after completing renal ablation for the duration of the study. The uraemia mouse model was set up successfully after twelve weeks, which was confirmed by a dramatic increase level of urea, creatinine and urine protein in 24 hours compared with the SOR group. Finally, the mice were sacrificed, and the thoracic aortas were dissected from the mice. qRT–PCR was used to measure the expression levels of miR-34b and western blot and immunohistochemistry analysis were used to test the expression of ALP, OC, Runx2, DNMT3a, BTG4 and Notch1 protein in aortic tissues. Alizarin Red S staining was used to detect artery calcification. Some of the artery samples were used for BSP to assess the methylation of miR-34b in aortic tissues. Total protein was quantified using the Bradford protein assay. The investigation conformed to the Guide for the Care and Use of Laboratory Animals published by the United States National Institutes of Health (NIH publication no. 85–23, revised 1996). The animal study was approved by the Ethics Committee of the Second Xiang-Ya Hospital, Central South University.

### Immunohistochemistry

Arterial samples were fixed and processed by the paraffin-embedded method. Arterial tissue sections were deparaffinised in xylene and rehydrated in a graded ethanol series. To clear endogenous peroxidase, sections were incubated with 3% hydrogen peroxide, followed by antigen retrieval with trypsin. After blocking with 5% BSA, slides were probed overnight at 4 °C with polyclonal antibodies against DNMT3a and Notch1. The primary antibody was detected by a biotinylated secondary antibody followed by the avidin-biotin peroxidase complex and 3,3'-diaminobenzidine chromogen (GK5007, GTvision, Shanghai, China). The positive results were measured using a Nikon Eclipse microscope with a Nikon DSR1 camera was analysed by Nikon NIS-Elements AR software (Nikon Instruments Korea Co, Ltd. Seoul, Korea).

### Patients and arterial tissue samples

A total of five pairs of renal artery segments from uraemia patients and normal healthy subjects were obtained from the Department of Urological Organ Transplantation of the Second Xiang-Ya Hospital, Central South University. The patients were those with ESRD who needed a kidney transplant, and the donors were those willing to donate their kidney to the patients with ESRD. The arterial tissues were collected during the process of the kidney transplantation surgery. Some of the samples were fixed with 4% paraformaldehyde and stored at room temperature, while others were frozen and stored in liquid nitrogen. The clinical study was approved by the Ethics Committee of Second Xiang-Ya Hospital, Central South University, and written informed consent was obtained from all participants in our experiments. The information regarding age, gender and group numbers of the patients were shown in Supplementary Table 2.

### Statistical analysis

The data were presented as mean ± standard deviation (SD) and the analysis was performed with GraphPad Prism software (GraphPad Prism version 6.0). The normality of data distribution was assessed before analysis, and the Student’s t-test was used to compare normally distributed data between two different groups. Comparisons of multiple groups were made using one-way analysis of variance (ANOVA). A level of *p<*0.05 was considered statistically significant. All experiments were repeated at least three times, and representative experiment results were shown in the figures.

## Supplementary Material

Supplementary Tables and Figures
