# Supplementary material for: Aberration methylation of miR-34b was involved in regulating vascular calcification by targeting Notch1
Source: Aging (Albany NY). 2019 May 25;11(10):3182–97. doi: 10.18632/aging.101973 (PMC6555467; doi:10.18632/aging.101973)
Supplement: Supplementary Tables and Figures [file aging-11-101973-s001.pdf]

SUPPLEMENTARY MATERIALS

Supplementary Table 1. Nucleotide sequences of primers of miR-34b for BSP.

Gene	Primer sequence(5′ to 3′)
Mmu-miR-34b	Sense: GTTTTAGATTTGGGTTTGGAAGT<br>Antisense: CAATAAAATTAATAATTATCAACACC
Has-miR-34b	Sense: AATGAGGGAGTGGAGGAGTTT<br>Antisense: ACCCCAAACCCTAAAACTAACT

Supplementary Table 2. Characteristics of the patients.

	Normal	ESRD
Age (years)	26.35 ± 3.235	43.42 ± 3. 25
Genders	Male: 4	Male: 3
	Female: 1	Female:2
Cre (umol/l)	56.35 ± 2.726	1098 ± 134.6*
BUN (mmol/l)	5.631 ± 0.456	31.2 ± 2.358*
UA (umol/l)	284.6 ± 18.46	466.2 ± 21.49*

Abbreviation: Cre: creatinine; BUN: blood urea nitrogen; UA: uric acid; *p<0.05, compared with Normal group.

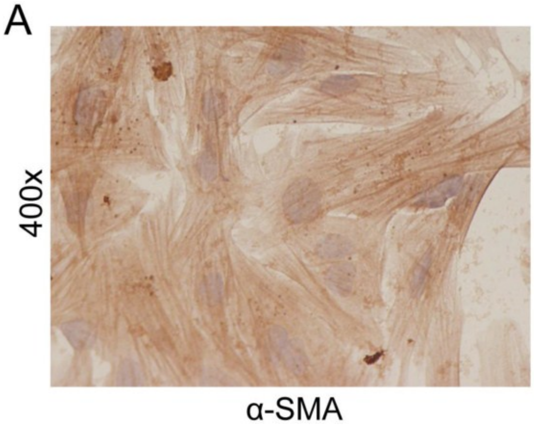

Supplementary Figure 1. Immunocytochemical staining was used to identify the phenotype of VSMCs by detecting the expression of a-SMA.

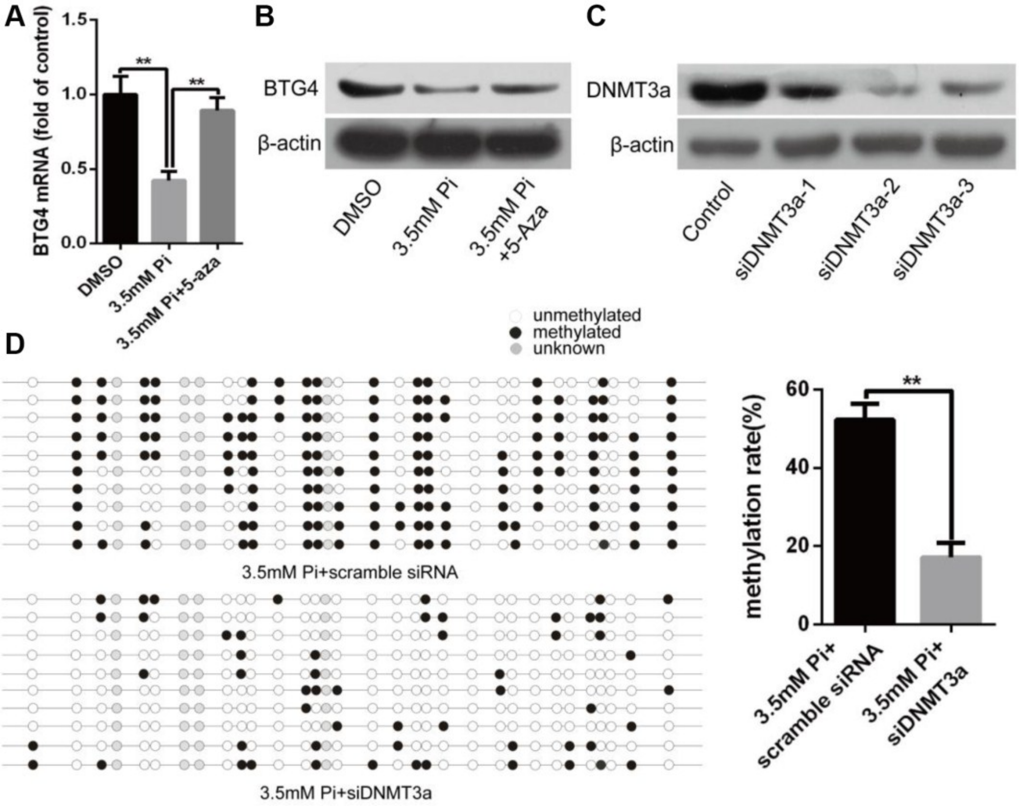

Supplementary Figure 2. (**A**) The expression of BTG4 mRNA detected by qRT-PCR in VSMCs treated with 3.5mM Pi or 3.5mM Pi + 10 μM 5-aza. (**B**) Western blot analysis showed that 5-aza could upregulate the level of BTG4 protein in VSMCs. (**C**) The inhibitory efficiency of siRNAs targeting DNMT3a was verified by western blot analysis. (**D**) BSP showed that the methylation rate of CpG sites of miR-34b DNA was significantly lower in VSMCs with 3.5mM Pi + siDNMT3a treatment than that of 3.5mM Pi + scramble siDNMT3a. N=3. The data are expressed as mean ± SD, *p <0.05;**p<0.005.

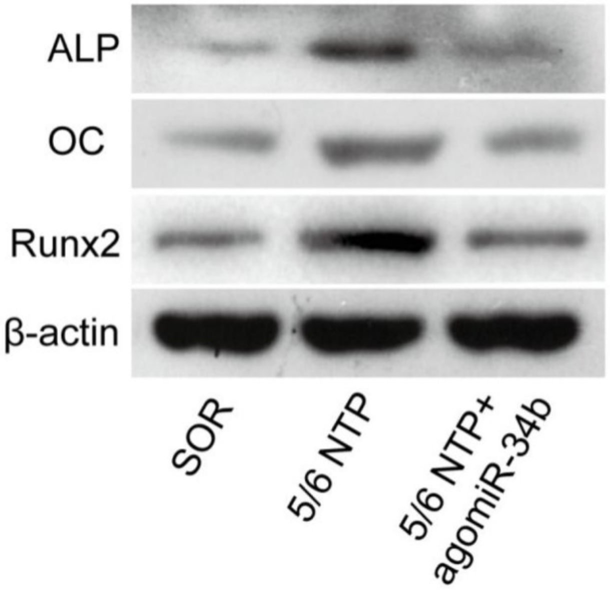

Supplementary Figure 3. Western blot analysis measured the level of ALP activity, OC secretion and Runx2 expression in arteries from SOR mice, 5/6 NTP mice and 5/6 NTP+agomiR-34b mice. N=5. SOR: sham operation; 5/6 NTP: 5/6 nephrectomy with a high-phosphate diet.
